# What is behind the gender gap in economics distance education: Age, work-life balance and COVID-19

**DOI:** 10.1371/journal.pone.0272341

**Published:** 2022-08-08

**Authors:** Cristina Castellanos-Serrano, Gonzalo Escribano, Juandiego Paredes-Gázquez, Enrique San-Martín González

**Affiliations:** Applied Economics Department, Universidad Nacional de Educación a Distancia (UNED), Madrid, Spain; Universidad Carlos III de Madrid Departamento de Matematicas, SPAIN

## Abstract

There is an ongoing debate about whether gender equality in education has been achieved or not. Research efforts have focused on primary and secondary education, while there are fewer studies on higher education, and few studies refer to distance education. To contribute to address this gap, this article presents a gender analysis of educational outcomes in economics at Spain’s leading distance university, UNED, which is also the largest university in the European Union in terms of enrolment. The aim of the article is to assess whether there is a gender gap in academic results and to identify the sociodemographic and academic variables that may be causing such a gap by analysing how they shape such differences. Finally, the impact of COVID-19 is also considered. The results confirm that women underperformed significantly in our sample in terms of passing and scoring, especially among those between 30 and 45 years of age, who are more likely to have young children. When considering a distribution of family tasks biased against women, along with the higher average age of distance learning university students, gender gaps could probably be greater in nonface-to-face education. COVID-19 narrowed the gender gap during the lockdown period, as some men and women staying at home together were able to improve task sharing capabilities. After the lockdown, however, women’s results worsened compared to pre-COVID-19 levels. A possible explanation is that they had to continue performing the same family duties in addition to substituting education and caring services (e.g., nurseries and day centres for the elderly) that did not resume activity immediately or continuously.

## 1. Introduction

There is an ongoing debate about whether gender equality in education has been achieved. Although men seem to perform better on standardized aptitude and achievement tests, women outperform men in scores on a whole subject or academic year [1–3]. Voyer & Voyer (2014), through a meta-analysis [2], found evidence suggesting that women perform slightly better than men at all stages of the education system and in all branches. However, there are so many exceptions and nuances to this evidence that they preclude reaching an unequivocal conclusion. Moreover, regular evidence on university outcomes from a gender perspective is not easily available. For instance, the UNESCO report [4] shows differences in primary and secondary school results, but university results are not presented. Additionally, distance and nonface-to face education is often overlooked or forgotten. This article aims to contribute to the gender debate in higher education with a focus on nonface-to-face education. This methodology is increasingly in demand, and events such as COVID-19 have highlighted its usefulness and importance. This article analyses data from students at UNED, the oldest distance-learning university in Spain and the largest by enrolment in the EU since 2013 [5, 6].

Generally, higher or tertiary education is thought of as a stage of the first part of adulthood in which young adults must make sense of who they are and who they will become in addition to obtaining certain knowledge and competencies [7]. Consequently, most evidence, especially that on the impacts of COVID-19 on higher education, mainly focuses on young students [8–12]. However, the UNED profile is different, with students considerably being older and who may have spent long periods out of formal learning and could have other university degrees and/or substantial work experience. Moreover, they tend to combine their studies with other responsibilities, such as paid work and caring for their families. Considering the vast literature on the unequal distribution of household production and care work between women and men [13, 14], it seems relevant to consider whether this factor may also impact on distance higher education as it does on labour market gender gaps [15]. Moreover, the COVID-19 shock has impacted the distribution of paid and unpaid work and total work hours across nations [16–23], and it may have also affected distance higher education results.

On the other hand, the particularities of distance learning universities with face-to-face exams, such as UNED, are not well known either, especially when variables such as sex, age and fields of knowledge are considered together. COVID-19 has also affected these universities, although not with the same intensity as traditional universities. However, the effects on learning outcomes may be even stronger as distance university students are different, more mature, and have other responsibilities in addition to their studies. Therefore, we also consider COVID-19 in our analysis.

Thus, our main objective is to assess whether there are observable gender gaps in the academic results in economics at a nonface-to-face university and to identify which factors may motivate these gaps. Variables related to sociodemographic and academic characteristics, as well as COVID-19 lockdown impacts, are analysed, as they may differentially affect the academic results of women and men. How these variables shape such differences between men and women is our secondary focus. Consequently, a heterogeneity analysis with variable interactions will be carried out, although this type of analysis, which considers sex and other variables at the same time, is better known in the gender literature as intersectionality. When considering the specificities of distance learning, our results present evidence of women underperforming in higher education. What are the reasons for these results? The article suggests that the unequal distribution of domestic and care tasks between men and women, especially for the middle-aged group (30–45), which mostly coincides with the UNED student profile, is a key factor in explaining such performance differences, as Richardson *et al*. pointed out more than twenty years ago [24].

The article is structured as follows. Section 2 reviews the literature on gender equality and higher education, including in relation to COVID-19 impacts. Section 3 presents the empirical design used, and Section 4 provides the results of the analysis. Section 5 discusses these results, and Section 6 concludes and proposes policy measures to reduce the gender gap in distance learning higher education.

## 2. Gender equality and higher education

### 2.1. Outlook and literature review

Although the debate regarding gender equality in education is ongoing, some researchers support the hypothesis that women generally outperform men in education results from elementary school to the university level [2]. However, not all women outperform: nationality, age, income and other factors may have a strong influence. UNESCO measured and recognized both facts in primary and secondary education [25], but gender intersectionality data are not systematically available for higher education. Voyer & Voyer [2] point out in their meta-analysis that this female advantage is small and that the nationality and gender composition of samples are significant moderators of effect sizes. Although women were more likely to obtain better scores, this trend did not occur at all ages or in all subjects [26].

In 2018, women accounted for more than half of 17.5 million tertiary students in the EU-27 [27]. Women are the majority in most fields except for some branches of STEM [4]. However, gender equality is measured not only by access but also by results, as occurs in the labour market. There is not a systematic measurement of higher education outcomes at the global level, although the EU and national systems are developing data disaggregated by sex [28, 29]. Within the EU-27, almost 60% of all graduates in 2018 were women, and graduates’ distribution in fields of education by sex are similar to those for enrolled students. Similarly, women tend to obtain slightly better scores. In Spain, the average scores for a bachelor’s degree in the 2019/20 academic year were 7.39 for women and 7.09 for men, similar to previous years [29]. The same occurs in the social sciences and economics, where our focus lies. Beyond scores, perceived and observed learning outcomes did not differ significantly by sex [30, 31].

Regarding e-learning, there are few differences between male and female students in their enrolment, motivation and satisfaction patterns, mainly regarding their interactions with technology [32]. There are no gender differences in student satisfaction among millennials [33], but beyond this age range, there is mixed evidence [31, 32, 34, 35]. Lu & Chiou [35] point out that gender and job status significantly influence students’ satisfaction and their perceptions about interface usability, community membership, content richness and flexibility. However, according to these authors, only job status and learning styles moderate the relationship between previous perceptions and satisfaction, while gender has no effect in e-learning environments.

Comparisons of online and on-campus settings show statistically significantly better online learning outcomes than traditional learning results [31]. Women were found to outperform men in academic achievement both online and on campus [36]. It seems that different motivational factors (self-determination, culture or study as a hobby, job opportunities and promotion, etc.) are influenced by sex and age, which are reflected in educational outcomes [37]. The positive relationship between sex and academic performance is explained when students are older, but deep learning is more likely deployed by older women [36]. Age itself does not predict adult students’ learning satisfaction and performance [38], but when women underperform, Richardson *et al*. [24] suggest that this may be because, within a particular age range, women usually combine occupational and domestic responsibilities.

However, some variables are missing from the literature on gender gaps in education. When nationality has been traditionally included in educational research, it has been mainly done for the analysis of the integration of immigrants in primary or secondary schools. On many occasions, students’ sex is also considered in analyses. However, when taking into account the age difference between average UNED and university students, our subjects of analysis are mainly adult individuals who work and study outside their countries of origin. Including this variable in the distance learning context could be an interesting novel approach, as we have not found references that deal with context.

Terms represent another interesting variable not explored in the literature. Perhaps this may be because in face-to-face education, with most students engaged in full-time study, the differences between terms are not significant. However, when students combine study, work and family life, as is the case in nonface-to-face education, fatigue may be a relevant factor. Our exploratory analysis shows significant differences between semesters, leading to their inclusion in the analysis.

### 2.2. COVID-19, higher education and domestic and care work: Does sex matter?

Over the course of the COVID-19 lockdown, tertiary students were forced to shift to online education worldwide. Previous research has focused on how the initial movement to online teaching among face-to-face universities due to measures against COVID-19 has widened socioeconomic educational gaps, even leading to widening inequality and increasing poverty in countries such as the US, Italy, Sweden and Turkey [8, 9, 11, 12]. Although many use sex as a control variable [8, 11, 12], these articles are not focused in the gender gap and gender intersectionality is not even considered. In contrast, Casalone et al. [10] apply a gender perspective while drawing national comparisons, bringing intersectionality into gender analysis. Age, sex, ethnicity, nationality and income level are among the key factors considered in the literature related to the effects of COVID-19 on tertiary education.

In Spain, passing rates and scores seem to have improved. Gonzalez *et al*. [39] attribute better student performance to a general change in the autonomous learning process, as assessment activities and learning methodologies cannot explain it. Students work with more adequate time management, creating positive results. On the other hand, students from families with a low educational level had fewer opportunities to use digital technologies during the COVID-19 lockdown [40]. Adaptation to online higher education depends on a set of factors, including institutional and pedagogical responses, individual self-regulatory and socioemotional competencies, and adequate resources [41].

However, COVID-19 has also affected higher education distance learning institutions. UNED had to shift examinations from face-to-face to online formats, which were temporarily maintained postlockdown until September 2021. The lockdown may have provided more time to study and improved results, as evidence suggests for Turkey and Italy [10]. In addition, men stayed at home more so they could carry out more tasks than usual [21]. However, we do not know if these changes are enough to compensate for difficulties caused by the disruption of the whole education system, from daycare and preschool centres to primary and secondary schools, which forced babies and children to stay at home. International comparisons suggest that forced isolation did not lead young women to neglect their studies to dedicate time to housework or childcare among those enrolled at on-campus universities that switched to online methods due to the pandemic [10]. However, the results may be not similar when most students are middle -aged, as is the case at UNED.

To our knowledge, the gender perspective has not been included in the analysis of COVID-19 effects on Spanish academic results, even when the sex variable has been available [39, 40]. Hence, this t analysis aims to partially overcome this gap in the literature regarding distance education. UNED academic results are analysed by considering the sex variable systematically with other factors that may contribute to explaining the gender differences observed. In particular, this research assesses whether the lockdown and the switch to online exams produced significant changes in students’ results and induced additional gender differences.

Moreover, given the age and labour status of UNED students, it is relevant to consider the gender inequalities observed before and during the COVID-19 crisis related to the distribution of paid and unpaid work. As with that focused on higher education, the literature on the effects of the COVID-19 crisis on the labour market, care and gender inequality [16–20, 22, 23] has mainly focused on the first months of this declaration, when the restrictions were the most severe. Among initial effects have been the large percentages of the employed population not working, a phenomenon more widespread among the less educated and blue-collar workers, and those working from home, especially among university-educated employees and white-collar workers, to a greater or lesser extent depending on the national context [20]. Similarly, an increase in the gender gap in total working hours is observed because the decrease in the number of hours of paid work does not compensate for the increase in time spent on unpaid work and the previous difference between men and women [19]. Women continued to bear a greater share of the burden of domestic work [16, 18, 19, 23], although men’s physical presence in the household helped slightly increase their participation in domestic and care work [16, 17, 23, 42]. This increased burden of unpaid work by women, even in an exceptional and critical situation such as the lockdown suffered in spring 2020, is independent of their labour market status [19, 22]. Men increased their involvement in domestic and care work, but the increased assumption of responsibilities did not lead to a generalized reduction of the gender gap in unpaid work [17–19, 22, 23], as the absence of educational services and the impossibility of outsourcing domestic and care work meant that many women also increased their participation in unpaid work.

This picture of gender inequality in paid and unpaid work remained consistent at least until June 2021 in Spain [21], where many conditions have been maintained to a greater or lesser extent from the lockdown to the present. In Spain, the second and subsequent waves of COVID-19 brought states of alarm until June 2021, which also entailed severe restrictions on mobility and contact with noncohabitants, maintaining strong encouragement to work from home, as well as partial or occasional restrictions in many educational centres, although educational services were theoretically resumed. At UNED, for example, exams remained online for 2020–2021.

## 3. Empirical design

### 3.1. Ethical approach to participants

In November 2021, UNED approved two nonfunded teaching innovation projects named “Inclusion of the gender perspective in teaching innovation” and “Improvements for academic performance in economic policy subjects after COVID-19” coordinated by two of the authors. These two projects form the basis of this article.

Although this research involved human participants, the study did not require ethics committee authorization according to UNED’s internal protocols, as it meets the three requirements established for this approval not to be needed:

- The research does not affect the fundamental rights (life, physical/psychic integrity, health, freedom/autonomy in any of its manifestations, personal dignity, etc.) of the subjects involved.
- Only nonidentifying personal data are used.
- Only data available for researchers on the quality of teachers are used.

The Vice-Chancellor for Research, Knowledge Transfer and Scientific Dissemination, who is also the president of the UNED ethics committee, certified this fact.

Data were anonymized by a random transformation of student IDs. Data collection did not include minors, and no personal data were gathered other than data on sex, age and nationality. These data were collected during the enrolment process, and students were informed that they may be used for the university’s purposes, including research. The participants may, at any time, exercise their rights of access, rectification, cancellation or opposition of their data, but they have not done so thus far. This research did not interfere with the activities, processes or assessments of the participating students in any way. Moreover, the teachers responsible for the subjects included in the study gave their verbal consent to carry out this research.

### 3.2. Gender equality in Spanish higher education: The UNED case

In Spain, women in higher education follow European trends. Women were the majority in undergraduate, master’s and doctoral studies in the 2019/20 academic year [29]. In the social sciences and law, women accounted for 60% of those enrolled and even 65% of those who finished their bachelor’s degree [43]. However, the student profile is clearly different for face-to-face universities than for nonface-to-face universities. For instance, in the social and legal sciences, in the 2018/19 academic year, almost 81% of enrolled students were under 25 years of age, and only 5% were over 30 years of age, while at UNED, only 16.04% of the students were under 25 [44]. Thus, it cannot be assumed that gender equality or gender gaps behave in the same way. Consequently, it is also interesting to analyse gender equality in nonface-to-face education.

Distance universities in Spain had 264,857 students enrolled, representing 16.2% of university students, in 2019/20 [29]. UNED is Spain’s national nonface-to-face public university and is the oldest distance education university in the country: in 2022, it celebrated its 50th anniversary. Since 2013, it has been the largest university in the EU by enrolment, both face-to-face and distance [5, 6]. In Spain, educational competencies are transferred to regions (autonomous communities), but UNED was founded in 1972, before the birth of autonomous communities, and has succeeded in remaining a national institution. In addition to UNED, there is only another national university, Menendez Pelayo International University, but it does not teach undergraduate courses. In the 2019/20 academic year, 157,418 students were enrolled at UNED [44], representing almost 60% of Spanish nonface-to face students. A total of 53.7% were women, and this level rose to 54.9% in 2020/21 [44]. From these numbers, it is clear that UNED students are a representative sample of distance education in Spain. Table 1 shows the gendered learning outcomes gap at UNED.

**Table 1:** Female participation and gender gap in educational outcomes by field of education at UNED (2016/17-2020/21).

Average data for academic years 2016/17 to 2020/21	Percentage of women enroled	Differences in evaluated students by sex in percentage points (women minus men)	Differences in passed students by sex in percentage points (women minus men)	Differences in average score (0–10) by sex (women minus men)
TOTAL UNED	55.1%	+5.7	**-2.2**	**-0.07**
Arts & Humanities	51.3%	**-0.2**	**-1.6**	**-0.04**
Psychology	71.0%	+1.7	**-2.6**	**-0.18**
Social Sciences	55.0%	+3.5	**-0.9**	**-0.01**
Faculty of Economics and Business	45.7%	+3.6	**-3.4**	**-0.19**
Faculty of Political Science and Sociology	37.5%	**-2.2**	**-4.0**	**-0.28**
Faculty of Law	54.2%	+0.7	**-3.3**	**-0.12**
Faculty of Education	80.0%	+4.8	+0.9	+0.06
STEM	25.3%	+5.5	**-0.6**	**-0.00**

Note: Negative numbers in bold: underperforming women. Source: Own elaboration based on UNED data [44]

As shown in Table 1, across UNED, women are enrolled in more subjects (55%) than men, and they tend to take exams in higher proportions (5.7% points–p.p.–more than men), but they pass at lower proportions (2.2 p.p.) although their scores are similar (slightly lower by 0.07). This trend is found in most fields with some exceptions. Education and STEM, which are the most female- and male-dominated subjects, respectively, present almost no gender gap in passing grades and scores. Psychology, economics and business, and political science and sociology present a higher gender gap in passing grades than arts and the humanities, STEM and even the social sciences as a whole. Therefore, is there a gender bias in these faculties?

To answer this question, the article focuses on the UNED Faculty of Economics and Business and, specifically, on a set of applied economics subjects described in the next section. This faculty and the examined subjects were chosen for several reasons. First, the faculty seems to have one of the largest gender gaps regarding enrolment, passing grades and scores of the university (see Table 1). While women tend to be the minority in economics departments [45, 46], more evidence is needed on the learning outcomes of gender gaps in economics. Second, the chosen subjects are drawn from similar fields of knowledge (economic policy and public finance), and have a similar evaluation system that facilitates the comparability of results. Third, none of the subjects are first-year subjects, and all but one are mandatory, which means that all students should have a basic knowledge of economics before enrolling and that all students must pass them to earn the degree. Finally, the authors of this article teach these subjects and, therefore, are allowed to access all academic and nonacademic data of their students in accordance with the protocols of the UNED ethics committee (see previous section).

In summary, considering the particularities of UNED, this research could be valuable for several reasons. First, the results do not necessarily correspond to the national average educational outcomes by sex, as our data are from a specific field of knowledge (applied economics) and from a nonface-to-face university. Second, UNED students are not necessarily young people who follow a specific training path but mostly adults with work and family responsibilities. In addition, the factors inherent to the UNED learning/teaching process could affect their results. UNED students choose this university because it affords them the opportunity to combine their studies with family (75%) or work obligations (87.8%) [47]. Thus, as middle-aged women usually assume more family responsibilities than their male counterparts, they are expected to show worse results. In contrast, deep learning–the attempt to understand the meaning and logic of course materials and its arguments and the opposite of memorization–is usually related to older students, although it is mediated by sex [36], so this can also be analysed. Third, although UNED is a distance learning university, it has been also been affected by the COVID-19 lockdown. The gender perspective has not yet been included in the analysis of COVID-19 effects on Spanish academic results.

### 3.3. Data and variables

The sample includes 7,477 UNED students of the Faculty of Economics Science and Business from the 2016/2017 to 2020/2021 academic years. The unit of analysis is enrolment by subject and student. The students in the sample applied for a total of 16,821 enrolments. The nine subjects selected are taught in only one semester, as is the most common in degrees at UNED. Subjects belong to two fields, economic policy (five subjects) and public finance (four subjects), taught in four degrees: economics (68%), business administration (20,6%), tourism (10,3%) and political sciences (1,1%). Table 2 shows the main academic features of the subjects, including their enrolment levels by academic year and the average percentage of women enrolled. As expected, the sample is male dominated (62.5% of students are men), except in the tourism program, for which the shares are reversed (67.5% women).

**Table 2:** Subjects and enrolment data of the sample.

Degree	Subjects	Year	Term	Type	Academic years					Total	% Women from total
					2016/2017	2017/2018	2018/2019	2019/2020	2020/2021		
Economics	Budget and public expenditure theory *	2	1	Mandatory	457	475	417	382	445	2176	29,1%
	Theory of public revenues	2	2	Mandatory	518	519	426	** 375		1838	30,1%
	Economic policy: objectives and instruments	3	1	Mandatory	345	307	312	262	345	1571	29,3%
	Public economic policies	3	2	Mandatory	406	328	346	** 293		1373	31,3%
	Budget and public spending in Spain	4	1	Mandatory	282	280	257	233	264	1316	39,9%
	Spanish and comparative economic policy	4	1	Mandatory	282	306	331	248	280	1447	30,1%
	Spanish tax system	4	2	Mandatory	418	497	515	** 275		1705	32,9%
Business Administration	Public economic policies	3	2	Mandatory	965	895	797	** 816		3473	42,3%
Tourism	Tourism economic policy	4	1	Mandatory	437	372	325	283	322	1739	67,5%
Political Science	Budget and public expenditure theory *	4	1	Optional	41	22	33	34	53	183	32,2%
	Total (9 subjects)				4151	4001	3759	3201	1709	16821	37,5%

Notes:

* This subject is taught in two degrees.

** Subjects taught during the COVID-19 lockdown.

Source: own elaboration based on Portal Estadístico (UNED Statistical Office, 2021)

Table 3 shows descriptions and measurements of the eleven variables, dependents and independents, used in the models. Data were collected exclusively from three different UNED internal sources:

- The dependent variables, which measure academic performance (evaluated, passed and scored), were obtained from the administrative database of the university.
- Most of the variables (sex, age, nationality, term and degree) were obtained from the enrolment process.
- The last three variables were collected from the university online learning platform (continuous assessment–CA–test and, forum messages) or constructed (COVID-19) by the authors.

**Table 3:** Description and measurement of the variables.

Dependent	Description	Values
Evaluated	Whether the student has taken the examination of the subject in ordinary exams.	0. Not evaluated
		1. Evaluated
Passed	Whether the student has passed the subject in ordinary exams.	0. Did not pass
		1. Passed
Score	Score achieved by the student in the subject	0 (min.) to 10 (max.)
Independent	Description	Values
Sex	Sex of the student	0. Man
		1. Woman
Age	Age of enrolment in a subject	Years
Nationality	Nationality of the student	0. Spanish
		1. Foreign
Term	Whether the subject taught out from October to February (first term) or from February to June (second term)	0. First term
		1. Second term
Degree	Degree of enrolment	0. Economics
		1. Business Administration
		2. Tourism
		3. Political Science and Administration
CA test	Whether the student has taken the CA test, regardless of the score obtained	0. Did not take the CA test
		1. Did take the CA test
Messages	Number of messages that the student has posted in subject forums	Number of messages
COVID-19	Distinguishes three time periods: pre-COVID-19, lockdown (February to June 2020) and after lockdown	0. Pre-COVID
		1. Lockdown
		2. After lockdown

Most of the variables shown in Table 3 are self-explanatory or are clearly specified in the table. However, some need further explanation to put them in the context of UNED and, in general, of nonface-to-face higher education.

All three dependent variables (evaluated, passed and score) measure learning outcomes and are commonly used in the literature. For instance, evaluated and passed are similar variables to withdrawal and failure rates, while scoring or grading is almost always considered, although the scoring systems used can vary [48]. The three variables are related somehow to exams. At UNED, final ordinary exams on each subject are taken at the end of the term (February and June), but there is also a major examination period for both terms in September. However, we focus on regular university terms, as their learning outcomes cover most of the academic year, they occur closer to the teaching period, and taking them into account together with the extraordinary results would make the analysis excessively complex. Once students are enrolled in a subject, they can either take the final exam and be evaluated correspondingly or not. They have a maximum of six opportunities in total to pass each subject. Thus, many of them do not take an exam even if they are enrolled if they believe that they are not sufficiently prepared or are unlikely to pass. In the end, when students do not take their exams, this is a failure and a waste of resources, both private and public, although we have no data to analyse the reasons this occurs (which may be related to work, personal reasons, or, medical or academic factors). An exam is scored on a scale of 0 to 10. When voluntary CA test is taken, its score (of between 0 and 1) is added to the exam grade. When overall score of 5 or higher is achieved, the student has passed the subject.

Regarding “terms” or semesters, Spanish university students traditionally enrol for a full academic year from October to September, including subjects taught in the first (October-February) and second (February-June) terms. Until recently, students could not freely enrol in February, as is found in other education systems. Even today, enrolling in February at UNED is conditional on having registered for a minimum number of credits or subjects in the first term. With the exception of official internships, all subjects at the UNED Faculty of Economics are taught independently in just one term. Subjects taught in the upper years are usually more complex than those taught in the first years, but, within the same year, the level of difficulty of the terms is similar. At UNED, most students enrol in second-term subjects not knowing their first-term results; they combine study, work and family, as is the case in nonface-to-face education, and finally, the Christmas holidays occur immediately before the first-term final exams. In contrast, in the second term, there are no days off before final exams. These three factors together could explain the worsening of learning outcomes in the second term, as disappointment and/or fatigue may affect the students. The difference between terms could be seen as a first indication of the higher drop-out rates of nonface-to-face higher education detected in the literature [49, 50].

When the COVID-19 lockdown was established in Spain (March 15, 2020 to June 21, 2020), distance learning centres such as UNED did not have to completely change their learning methodologies, although some adjustments had to be made. For instance, at UNED, noncompulsory, complementary face-to-face group mentoring and face-to-face examinations went online. Additionally, the change in the way exams were taken also led to a loss of flexibility in combining studies with other responsibilities, such as work or family. Before COVID-19, exams for each subject were held at UNED centres across the country and abroad in morning and evening shifts in two different weeks. Students could opt for either shift without having to ask or inform anyone. When the university was forced to switch to online exams, students were assigned by surname to one of the shifts so as not to overload university servers. Although a change in shift could be requested, there had to be a justification, and this had to be done in advance, eliminating the flexibility to adapt to unforeseen events that are common when caring for family members. Therefore, UNED students have to adapt to the lockdown measures, not only outside but also within the university, and it is relevant to study whether gender issues affected this adaptation through a comparison of the pre-COVID-19, lockdown and postlockdown periods.

### 3.4. Sample description

Table 4 presents descriptive statistics of both the dependent and independent variables. They are separated into different panels according to the type of variable (categorical in Panel I and continuous in Panel II). Most students did not take the exams and were not evaluated. Although women were evaluated in a slightly higher proportion than men (49.5% vs. 47.4%), they had worse academic results, as 42% of women did not pass in comparison to 33.3% of men, and women had an average score of 4.81 while men had an average score of 5.3.students are older than the national average, as explained in subsection 3.2. Only 4.3% of the students are foreigners, enrolment is balanced between the terms. Men and women participated almost equally in the CA test and in forums, although most of them dod not take the CA test (71.2%), and the average number of messages by a student was very low (0.38). The correlations table, which shows no relevant correlations, is presented in Table 5.

**Table 4:** Descriptive statistics.

Panel I. Categorical variables				
		Sex		
		Men	Woman	Total
Evaluated	Did not evaluate	5527	3190	8717
		52.6%	50.5%	51.8%
	Evaluated	4978	3126	8104
		47.4%	49.5%	48.2%
	Total	10505	6316	16821
Passed	Dit not pass	1657	1314	2971
		33.3%	42.0%	36.7%
	Passed	3321	1812	5133
		66.7%	58.0%	63.3%
	Total	4978	3126	8104
Nationality	Spanish	10237	5865	16102
		97.4%	92.9%	95.7%
	Foreign	268	451	719
		2.6%	7.1%	4.3%
	Total	10505	6316	16821
Term	First term	5130	3302	8432
		48.8%	52.3%	50.1%
	Second term	5375	3014	8389
		51.2%	47.7%	49.9%
	Total	10505	6316	16821
Degree	Economics	7813	3613	11426
		74.4%	57.2%	67.9%
	Business Admin.	2003	1470	3473
		19.1%	23.3%	20.6%
	Tourism	565	1174	1739
		5.4%	18.6%	10.3%
	Political Sci.	124	59	183
		1.2%	0.9%	1.1%
	Total	10505	6316	16821
Continuous assessment (CA) test	No CA test	7599	4381	11980
		72.3%	69.4%	71.2%
	CA test	2906	1935	4841
		27.7%	30.6%	28.8%
	Total	10505	6316	16821
COVID-19	Pre COVID-19	8324	5029	13353
		79.2%	79.6%	79.4%
	Lockdown	1133	626	1759
		10.8%	9.9%	10.5%
	After lockdown	1048	661	1709
		10.0%	10.5%	10.2%
	Total	10505	6316	16821
Panel II. Continuous variables				
	N	Min-Max	Average	Std. dev.
Score	8104	0–10	5.113	2.488
Men	4978	0–10	5.3	2.5
Women	3126	0–10	4.81	2.44
Age	16766	19–89	35.94	10.074
Men	10482	19–85	36.53	10.54
Women	6284	19–89	34.95	9.17
Messages	16821	0–70	0.38	1.945
Men	10505	0–70	0.37	2.07
Women	6316	0–49	0.38	1.72

**Table 5:** Correlations.

	Evaluated	Passed	Score	Sex	Age	Nationality	Term	Degree	CA Test	Messages
Passed	-^e^	1								
Score	-^e^	-^e^	1							
Sex	-0.032^d^	0.142^d^	0.121^b^	1						
Age	-0.011^b^	0.149^b^	0.125^a^	0.100^b^	1					
Nationality	0.004^d^	0.074^d^	0.086^b^	0.296^d^	0.133^b^	1				
Term	0.190^d^	0.012^d^	0.052^b^	-0.053^d^	-0.005^b^	-0.019^d^	1			
Degree	0.085^c^	-0.066^c^	-0.068^b^	-0.296^c^	0.040^b^	-0.091^c^	-0.040^c^	1		
CA Test	-0.607^d^	-0.331^d^	-0.293^b^	0.053^d^	-0.042^b^	-0.069^d^	0.005^d^	0.131^c^	1	
Messages	0.000^b^	-0.001^b^	-0.014^a^	0.005^d^	-0.003^a^	0.011^b^	-0.017^b^	0.010^b^	-0.001^b^	1
COVID-19	0.000^c^	-0.047^c^	-0.013^b^	0.002^c^	0.034^b^	0.052^c^	0.164^c^	0.019^c^	-0.273^c^	-0.015^b^

Notes: The type of correlation depends on the type of variable and it is as follows:

^a^. Pairwise correlation;

^b^. Polyserial correlation;

^c^. Polychoric correlation;

^d^. Tetrachoric correlation;

^e^. Non-computable correlation.

### 3.5. Models

The method chosen to estimate the models is contingent on the dependent variable. On the one hand, if the dependent variable is binary (evaluated and passed), we use a Logit model. Logit models were estimated by maximum likelihood. This method estimates the parameters maximizing the probability of obtaining the observed data [51]. In these cases, the effect of the independent variables is provided, including log-odds or logits and odds ratios. The. T percentage change in the odds is also included in the supplementary information. On the other hand, for the continuous dependent variable score, we estimate an ordinal least squares (OLS) multiple linear regression model.

Since our data are cross-sectional (students do not remain in all the courses in the sample), but students can enrol several times in one subject or in more than one subjetc at the same time, we used population-averaged (pooled) models to estimate the parameters [52]. In both logit and regression models, the standard errors are corrected with cluster-robust standard errors by student ID to control for heteroskedasticity [53], since a student can enrol in one subject several times. In addition, for each categorical variable, we set a base category, the one with the greater sample size (men, Spanish, the first term, economics, no CA test and the pre-COVID-19 period), as a reference for interpreting the log odds, the odds ratio, and the coefficients of categorical variables. This is why some values, the base categories, do not appear in all the tables or figures.

The objectives of this research are twofold: first, we seek to confirm or reject the existence of a gender gap in higher education in economics at the student level at a distance university, and second, we wish to identify how the independent variables shape differences between men and women in the dependent variables. This is why we include interactions in the design of the models. Interactions model how the coefficient for one variable differs according to the values of another variable [54]. In our research, we hypothesize that the effect of sex on the dependent variables varies depending on up to four independent variables (age, nationality, term and COVID-19). Interactions are included in the models by computing the product of two independent variables, which are also included as main effects. For example, a model including the interaction between sex and terms would include the two main effects (sex and terms separately) as well as the interaction (sex*term).

To address potential multicollinearity problems, the eight independent variables are divided into three groups: sociodemographic, academic and COVID-19. The first group of sociodemographic variables includes sex, age and nationality (Spanish or foreigner). The second group includes the academic variables: term (first or second term), degree (the four aforementioned degrees), continuous assessment test and student messages in forums. For each dependent variable, three nested models are estimated following a nested estimate procedure [55], which allows for detecting and reducing multicollinearity problems between the independent variables. The first models include only the three sociodemographic variables. The second models, or whole-single models, include all the independent variables but only their main effects. Finally, the third models, or whole models with interactions, include the eight dependent variables, main effects, and interactions of key dependent variables. The three nested models can be outlined as follows:

1. Dependentvariables=f(sociodemographicvariables).
2. Dependentvariables=f(sociodemographicvariables+maineffectsofindependentvariables).
3.
Dependentvariables=f(sociodemographicvariables+maineffectsofindependentvariables+interactionofkeydependentvariables).

The mathematical formula of the estimated models is shown in Eq 1.


$$depvar_{i}=\alpha +\beta_{1}sex_{i}+\beta_{2}age_{i}+\beta_{3}sex*age_{i}+\beta_{4}nationality_{i}+\beta_{5}sex*nationality_{i}+\beta_{6}term_{i}+\beta_{7}sex*term_{i}+\beta_{8}degree_{i}+\beta_{9}CA\;test_{\text{i}}+\beta_{10}messages_{i}+\beta_{11}COVID_{i}+\beta_{12}sex*COVID_{i}+\varepsilon_{i}$$ (1)

We ease the interpretation of the results by providing predictive margins for the interactions included in the models. A predictive margin is a postestimation statistic computed from predictions made from a model while some of the covariates are not fixed [56]. The contrast of predictive margins is a postestimation test that measures and tests the significance of the differences between the predictive margins of men and women. All analyses were conducted in Stata statistical software [57].

## 4. Results

**Fig 1:**
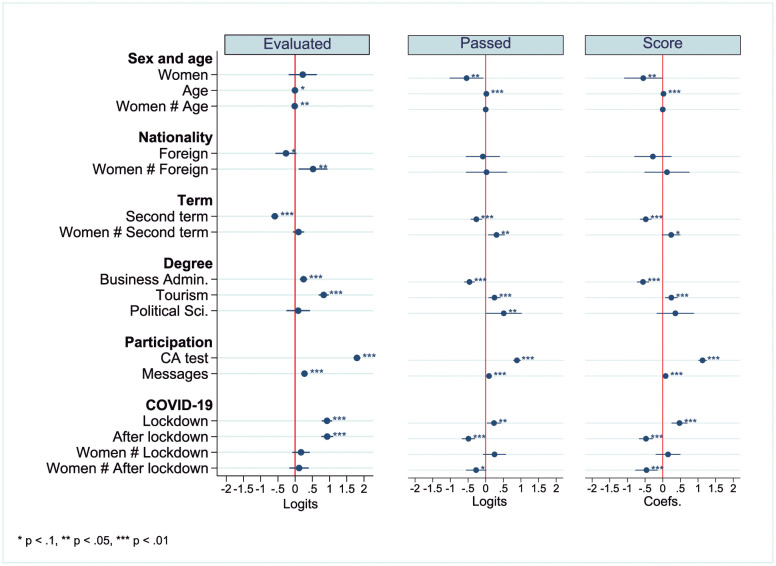
Graphical representation of the whole model with interaction estimation results.

Fig 1 shows a graphical summary of the results of the estimations for the three whole models with interactions. In this figure, all the sociodemographic, academic and COVID-19 variables’ main effects are significant in the three whole models with interactions, with the exception of sex (women) in the evaluated model, nationality in the passed and score models and the political science degree in the evaluated and score models. Regarding interactions, women interactions with age and nationality are not significant in the passed and score models; interactions with the lockdown are not significant in any of the three models, and interactions with terms and the postlockdown period are not significant in the evaluated model. To facilitate the interpretation of the results, the analysis is limited to the whole model with interactions, separating main effects, interactions and COVID-19. However, in the supporting information section, the complete set of nested models is shown in S1–S3 Tables.

**Fig 2:**
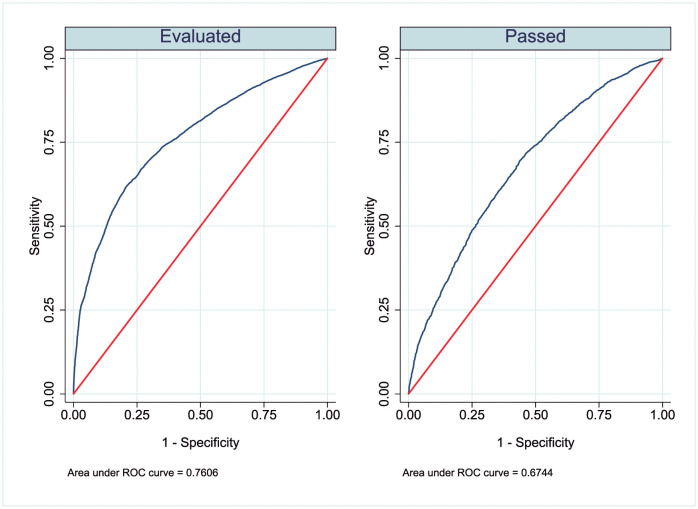
Receiver operating characteristic curves of the logit models.

To evaluate the performance of the two whole Logit models with interactions, we estimated their receiver operating characteristic (ROC) curves (Fig 2). The ROC curve plots sensitivity (true positive rate) against 1specifity (false- positive rate). The area under the ROC curve (AUC) provides an overall measure of the fit of the model, with values ranging from 0.5 (no discrimination power) to 1 (perfect discrimination). The evaluated model shows greater sensitivity than the passed model, with the first model presents a higher AUC value (0.760) than the second (0.674).

### 4.1. Main effects

The main effects results are shown in Table 6. Regarding **sociodemographic variables**, age is a statistically significant variable for the three whole models, although the effect is small (the odds ratio and coefficient are significant, but the value is very close to zero). The nationality odds ratio is statistically significant only in the evaluated model. The odds of evaluation decrease by a factor of 23.4% for foreigners. For sex, the main variable of this article, Table 6 shows that gender differences are not statistically significant in regard to taking exams (evaluated model), but women underperform significantly, as they passed less often than men and had lower scores. Women’s odds of passing decrease by 41.8%, and they are predicted to score 0.552 fewer points (5.52% as the score ranges from 0 to 10) than men. Therefore, in relation to our first objective, we can confirm that women underperform significantly in our sample, especially in terms of the likelihood of passing exams.

**Table 6:** Selected estimation results of the whole model with interactions: Main effects.

	Evaluated	Passed	Score
	O.R.	O.R.	Coef.
Women	1.248	**0.582** **	**-0.552** **
	(0.830–1.875)	(0.361–0.937)	(0.275)
Age	**0.995** *	**1.019** ***	**0.021** ***
	(0.989–1.001)	(1.011–1.026)	(0.004)
Foreign	**0.766** *	0.922	-0.279
	(0.567–1.037)	(0.570–1.493)	(0.267)
Second Term	**0.555** ***	**0.767** ***	**-0.481** ***
	(0.501–0.614)	(0.655–0.899)	(0.084)
Degree_Business Admin.	**1.278** ***	**0.632** ***	**-0.559** ***
	(1.147–1.424)	(0.545–0.732)	(0.088)
Degree_Tourism	**2.292** ***	**1.283** ***	**0.243** ***
	(1.980–2.653)	(1.083–1.519)	(0.090)
Degree_Political Sci.	1.092	**1.670** **	0.358
	(0.776–1.539)	(1.006–2.775)	(0.270)
CA Test	**6.028** ***	**2.426** ***	**1.128** ***
	(5.455–6.660)	(2.185–2.693)	(0.060)
Messages	**1.313** ***	**1.100** ***	**0.082** ***
	(1.225–1.406)	(1.054–1.147)	(0.011)
Lockdown (COVID)	**2.525** ***	**1.267** **	**0.474** ***
	(2.170–2.937)	(1.038–1.547)	(0.115)
After lockdown (COVID)	**2.541** ***	**0.614** ***	**-0.476** ***
	(2.143–3.012)	(0.509–0.740)	(0.101)
Constant	0.655***	0.844	4.247***
	(0.514–0.836)	(0.623–1.142)	(0.171)
N	16766	8082	8082
Ll	-9673	-4948	
R-squared			0.109
r2_a			0.107
chi2	2176	595.1	
F			54.07
df_model	16	16	16
P	0.000	0.000	0.000
N_clusters	7449	4544	4544

Notes: Robust standard errors are in parentheses;

*** p< 0.01,

** p< 0.05,

* p< 0.1.

Data in bold are significant variables The models include interactions, but their results are reported in Tables 7 & 10. The complete results of the models are provided in the Supporting information section.

Regarding academic variables, the term, the field of the bachelor’s degree and the participation variables (CA test and messages) are statistically relevant to predicting the odds ratio of being evaluated and passing and the final score. Moreover, relevant effect sizes are registered.

The term in which the subject is taught is statistically significantly related to being evaluated, to passing and to the obtained score. Academic results are expected to be lower in the second term. Students reduce their odds of being evaluated by 44.5%, and these odds decrease by 23.3% in the second term. The third model shows that scores of the second term are lower than those of the first term by -0.481 points.

Regarding degrees, the odds of evaluation are predicted to increase 1.278 times in business administration and 2.292 times for tourism in comparison to economics. However, students of business administration have lower odds of passing an exam (-36.8%) than students of economics, and their scores are expected to be lower by 0.559, as shown in Table 6. Students of tourism and political science show increased odds of passing (28.3% and 67%, respectively) relative to students of economics, but only in tourism is the score expected to be higher (0.243 points).

The participation variables (CA test and messages in forums) are statistically significant in all models as expected. Participation means that students actively follow the course and, consequently, the probabilities of taking an exam, passing and obtaining a good grade are much higher than those who do not. Since taking a CA test contributes to one’s final grade, the increase in the odds of any dependent variable is greater than that of participation in forums (messages).

COVID-19 restrictions have also impacted academic results. Both the lockdown period and subsequent movement restrictions positively and significantly impacted being evaluated with a relevant size effect (152.5% odds increase for the lockdown period and 154.1% odds increase for the postlockdown period). However, higher odds of passing (change in odds by 26.7%) and achieving a higher score (of 0.474 points) are predicted only for the lockdown period. After the lockdown periods, when some movement restrictions were still in place, academic results were expected to be worse due to the decrease in odds of passing (reduced by 38.6%) and lower scores (a reduction of 0.476 points) than in the period prior to the COVID-19 pandemic.

### 4.2. Intersectionality: Heterogeneity analysis within the gender perspective

**Fig 3:**
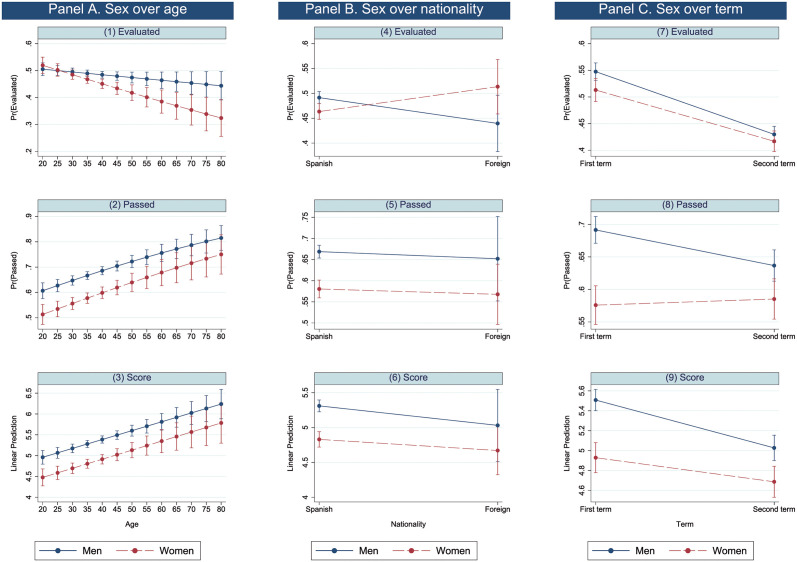
Predictive margins and linear predictions by sex.

Having dealt with the main objective of the article in the previous subsection, this subsection analyses not only how sex affects academic results per se but also how it affects such results considering its interaction with other variables to carry out a gender intersectional analysis. The relation between sex and the COVID-19 situation is treated separately. Finally, due to statistical parsimony, some interactions (degrees, CA testing and messages) were excluded in the definitive model. Fig 3 shows graphically the results of the gender intersectional analysis of the three whole models with interactions in Panels A (sex and age), B (sex and nationality) and C (sex and term), while Table 7 shows the quantitative results of the analysis.

**Table 7:** Selected estimation results in the whole model with interactions: Sex interactions.

	Evaluated	Passed	Score
	O.R.	O.R.	Coef.
Women	1.248	**0.582** **	**-0.552** **
	(0.830–1.875)	(0.361–0.937)	(0.275)
Age	**0.995** *	**1.019** ***	**0.021** ***
	(0.989–1.001)	(1.011–1.026)	(0.004)
Foreign	**0.766** *	0.922	-0.279
	(0.567–1.037)	(0.570–1.493)	(0.267)
Second Term	**0.555** ***	**0.767** ***	**-0.481** ***
	(0.501–0.614)	(0.655–0.899)	(0.084)
Women#Age	**0.988** **	1.000	0.000
	(0.977–0.998)	(0.988–1.013)	(0.007)
Women#Foreign	**1.684** **	1.024	0.120
	(1.111–2.555)	(0.572–1.835)	(0.325)
Women#Second Term	1.103	**1.359** **	**0.239** *
	(0.936–1.299)	(1.075–1.718)	(0.132)
Constant	**0.655** ***	0.844	**4.247** ***
	(0.514–0.836)	(0.623–1.142)	(0.171)
N	16766	8082	8082
ll	-9673	-4948	
R-squared			0.109
r2_a			0.107
chi2	2176	595.1	
F			54.07
df_model	16	16	16
p	0.000	0.000	0.000
N_clusters	7449	4544	4544

Notes: Robust standard errors in parentheses;

*** p< 0.01,

** p< 0.05,

* p< 0.1.

Data in bold are significant variables. Complete results of the models in the Supporting information section.

Graphically, there are significant differences if the standard deviation error bars of the categories of the sex variable do not overlap. As can be seen in Fig 3, there are significant sex differences for some ages in the three models of the sex and age interaction (Fig 3, A1-A3), for Spaniards in the passed and score models of the sex and nationality interaction (Fig 3, B5 & B6) and for the first term in the passed and score models of the sex and term interaction (Fig 3, C8 & C9). Finally, there is also a significant difference for the second term in the sex and term interaction of the score model (Fig 3, C9).

Quantitatively, a significant interaction term enhances, neutralizes or offsets the main effects of the variables included in the interaction. The age main effect shows a slight decrease (0.5%) in the odds of being evaluated. As women age, their odds of evaluating also decrease by 1.2%, as the increasing gap between men and women shows in Fig 3, A1. Women who take their exams in the second semester have 35.9% higher odds of passing once the negative main effects of being a woman (41.8% rate of decrease in odds of passing) and taking an exam in the second semester (23.3% rate of decrease in odds of passing) are considered. Thus, in this case, the interaction term partially offsets the main effects since the interaction increases the odds of passing and the main effects decrease them. This explains why the difference between men and women in the second term is smaller than that of the first term in Fig 3, C8. The same occurs for foreign women; the interaction term partially offsets the main effects, reducing the gap between men and women (Fig 3, B5).

**Fig 4:**
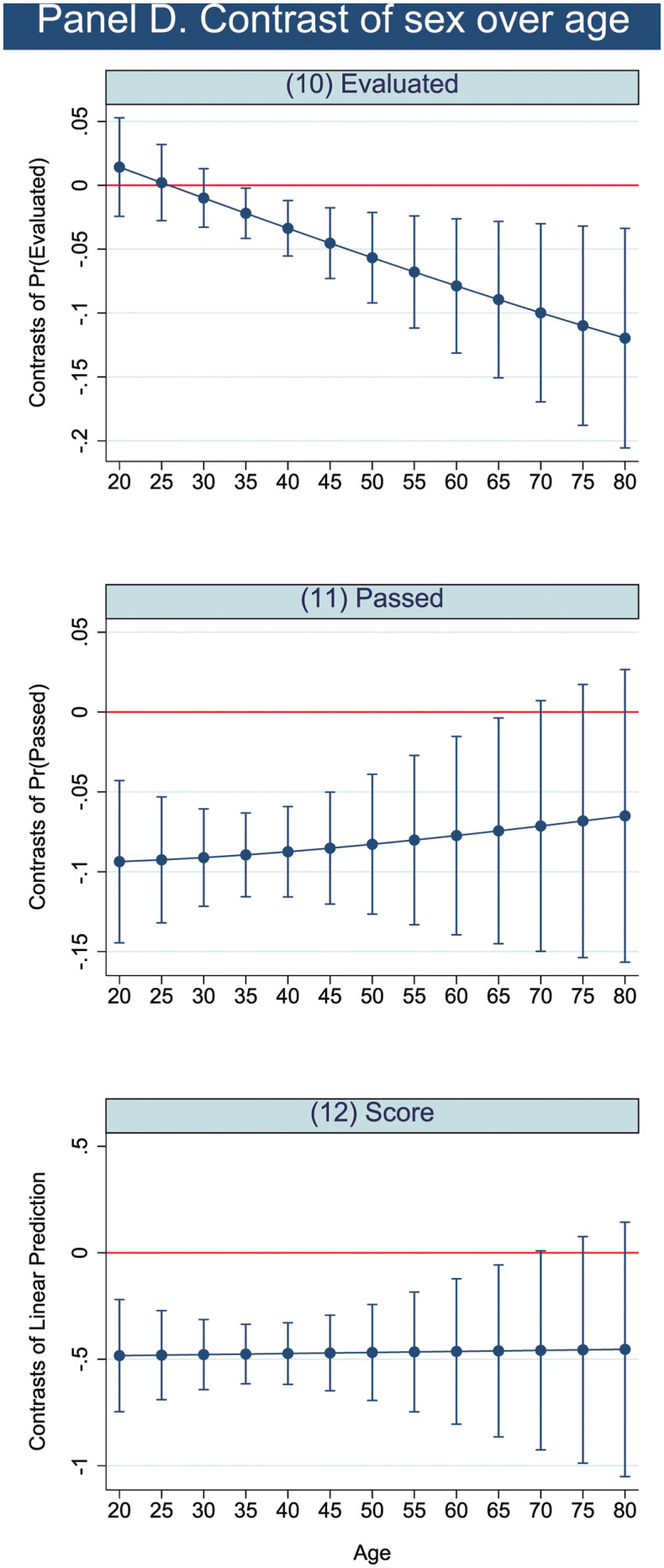
Contrast of predictions. Sex and age interactions.

Considering **sex and age** jointly, there are relevant differences between men and women in different age groups, especially regarding predictions of passing and scoring, as shown in Fig 3, A2 & A3. As we think that this interaction is the most important one, the differences between men and women are represented graphically in Fig 4 in addition to Table 8. In Fig 4 differences between men and women are significant if the standard deviation error bars do not cross the zero line. Men 40 years of age tended to be evaluated more than their female counterparts, although the difference was small (Fig 3, A1 and Table 8). No statistically significant differences are found under the age of 40. However, men of 20 to 70 years of age are expected to outperform women in terms of passing exams (Fig 3, A2 and Table 8) and scores (Fig 3, A2 and Table 8), with greater differences found between the ages of 30 and 45 (Fig 3, A2 & A3 and Table 8).

**Table 8:** Contrast of predictions. Sex and age interactions.

	Evaluated			Passed			Score		
	chi2	df	Contrast	chi2	df	Contrast	F	df	Contrast
Sex over age									
(Women vs Men) 20 years old	0.53	1	0.014	**13.07** ***	**1**	**-0.094**	**12.91** ***		**-0.483**
			(0.02)			(0.026)			(0.134)
(Women vs Men) 25 years old	0.02	1	0.002	**21.17** ***	**1**	**-0.093**	**20.26** ***		**-0.481**
			(0.015)			(0.02)			(0.107)
(Women vs Men) 30 years old	0.72	1	-0.01	**34.30** ***	**1**	**-0.091**	**32.31** ***		**-0.478**
			(0.012)			(0.016)			(0.084)
(Women vs Men) 35 years old	**4.72** **	**1**	**-0.022**	**44.59** ***	**1**	**-0.089**	**44.40** ***		**-0.476**
			(0.01)			(0.013)			(0.071)
(Women vs Men) 40 years old	**9.21** ***	**1**	**-0.034**	**36.65** ***	**1**	**-0.087**	**41.00** ***		**-0.473**
			(0.011)			(0.014)			(0.074)
(Women vs Men) 45 years old	**10.28** ***	**1**	**-0.045**	**22.75** ***	**1**	**-0.085**	**27.11** ***		**-0.471**
			(0.014)			(0.018)			(0.09)
(Women vs Men) 50 years old	**9.82** ***	**1**	**-0.057**	**13.75** ***	**1**	**-0.083**	**16.57** ***		**-0.468**
			(0.018)			(0.022)			(0.115)
(Women vs Men) 55 years old	**9.17** ***	**1**	**-0.068**	**8.78** ***	**1**	**-0.08**	**10.51** ***		**-0.466**
			(0.022)			(0.027)			(0.144)
(Women vs Men) 60 years old	**8.62** ***	**1**	**-0.079**	**5.96** **	**1**	**-0.077**	**7.06** **		**-0.463**
			(0.027)			(0.032)			(0.174)
(Women vs Men) 65 years old	**8.20** ***	**1**	**-0.089**	**4.26** **	**1**	**-0.074**	**5.00** *		**-0.461**
			(0.031)			(0.036)			(0.206)
(Women vs Men) 70 years old	**7.88** ***	**1**	**-0.1**	**3.18** *	**1**	**-0.071**	**3.69** *		**-0.458**
			(0.036)			(0.04)			(0.239)
(Women vs Men) 75years old	**7.63** ***	**1**	**-0.11**	2.45	1	-0.068	2.82		-0.456
			(0.04)			(0.044)			(0.272)
(Women vs Men) 80 years old	**7.44** ***	**1**	**-0.12**	1.94	1	-0.065	2.21		-0.454
			(0.044)			(0.047)			(0.305)
Joint	12.28	4		45.14***	4		22.74***		

Notes: Robust standard errors in parentheses;

*** p< 0.01,

** p< 0.05,

* p< 0.1.

Data in bold are significant variables.

Regarding **sex and nationality** (Table 9), there are almost no expected differences between men and women in terms of being evaluated, although Spanish women tend to be evaluated less than men, while foreign-born women tend to have a higher likelihood of being evaluated. Although the contrast shows a statistically significant adverse effect for Spanish women versus Spanish men, the difference in the predictive margins is small in the evaluated model (-0.028) and passed model (-0.088). Regarding the score model, the difference is similar to its main effect (-0.481 points). On the other hand, there is no statistically significant gender difference for foreign-born students regarding passing and scoring, but there is for evaluation, with women exhibiting a small positive difference in this case (0.080).

**Table 9:** Contrast of predictions. Sex interactions.

	Evaluated			Passed			Score		
	chi2	df	Contrast	chi2	df	Contrast	F	df	Contrast
Sex over nationality									
(Women vs Men) Spanish	**7.53** ***	**1**	**-0.028**	**43.13** ***	**1**	**-0.088**	**44.42** ***	**1**	**-0.481**
			(0.010)			(0.013)			(0.072)
(Women vs Men) Foreign	**3.83** *	**1**	**0.080**	1.72	1	-0.085	1.17	1	-0.342
			(0.041)			(0.064)			(0.316)
Joint	11.56***	2		44.51***	2		22.67***	2	
Sex over term									
(Women vs Men) First term	**6.87** ***	**1**	**-0.035**	**57.35** ***	**1**	**-0.131**	**54.77** ***	**1**	**-0.655**
			(0.013)			(0.017)			(0.088)
(Women vs Men) Second term	1.09	1	-0.011	3.51	1	-0.0316	**7.02** ***	**1**	**-0.241**
			(0.011)			(0.016)			(0.091)
Joint	6.94**	2		57.72***	2		27.85***	2	

Notes: Robust standard errors in parentheses;

*** p< 0.01,

** p< 0.05,

* p< 0.1.

Data in bold are significant variables.

Additional information of the contrast is available in S4 Table.

Regarding **term and sex** jointly (Table 9), there is a slight and statistically significant difference between the predictive margins of being evaluated in the first semester (-0.035). However, no difference is found in the second term. Men are expected to outperform women with higher predictive margins in the first term measured both by passing exams and scores. The difference between predictive margins of passing is statistically significant in the first term (-0.131), while it is not in the second term. Regarding scores, in the first term, women are predicted to obtain fewer 0.655 points than men, whereas their underperformance in the second term is less significant, as it is expected to be -0.241.

### 4.3. COVID-19 and sex interaction

**Fig 5:**
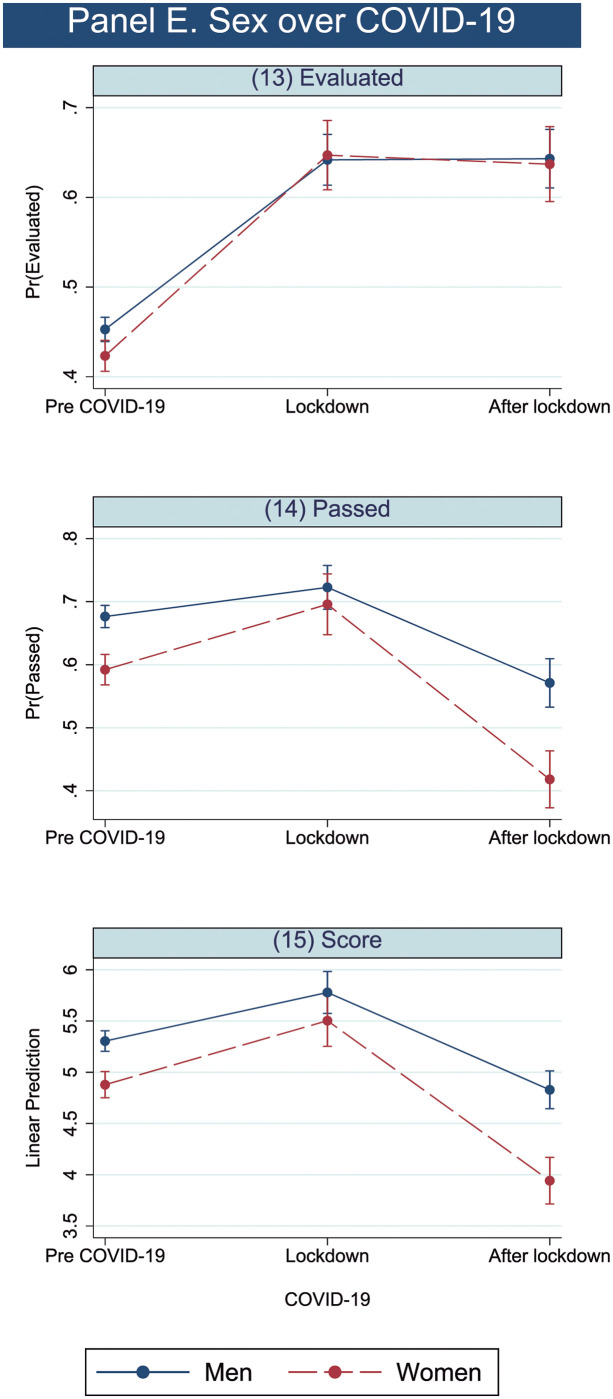
COVID-19 predictive margins and linear predictions by sex.

Table 10 shows the estimation results of the interaction between COVID-19 and sex. Regarding the passed model main effects, as stated above, the odds of passing decreased for women (reduction of 41.8%) as well as for exams done after lockdown (reduction of 38.6%), while these odds increased during the lockdown by 26.7%. Since the odds ratio of the interaction between women and the period after the lockdown has a value of below 1, women’s odds of passing further decrease by 23.8% once the negative effects of both main effects are considered, as the increasing gap between men and women shows in Fig 5, E11).

**Table 10:** Selected estimation results in the whole model with interactions. COVID-19 and sex interaction.

	Evaluated	Passed	Score
	O.R.	O.R.	Coef.
Women	1.248	**0.582** **	**-0.552** **
	(0.830–1.875)	(0.361–0.937)	(0.275)
Lockdown (COVID)	**2.525** ***	**1.267** **	**0.474** ***
	(2.170–2.937)	(1.038–1.547)	(0.115)
After lockdown (COVID)	**2.541** ***	**0.614** ***	**-0.476** ***
	(2.143–3.012)	(0.509–0.740)	(0.101)
Women#Lockdown	1.187	1.285	0.150
	(0.922–1.529)	(0.928–1.778)	(0.179)
Women#After lockdown	1.122	**0.762** *	**-0.461** ***
	(0.848–1.485)	(0.572–1.015)	(0.162)
Constant	0.655***	0.844	4.247***
	(0.514–0.836)	(0.623–1.142)	(0.171)
N	16766	8082	8082
ll	-9673	-4948	
R-squared			0.109
r2_a			0.107
chi2	2176	595.1	
F			54.07
df_model	16	16	16
p	0.000	0.000	0.000
N_clusters	7449	4544	4544

Notes: Robust standard errors in parentheses;

*** p< 0.01,

** p< 0.05,

* p< 0.1.

Data in bold are significant variables. Complete results of the models in the Supporting information section.

The results of the score model are similar to those of the passed model. First, both main effects coefficients, women and the period after the lockdown, show a negative effect on the score, while lockdown has a positive effect. Second, the interaction coefficient (women after the lockdown) is negative and significant, evidencing that women after the lockdown exhibited an additional decrease in the score to add to the negative effects of both main effects. Thus, the gap between men and women increased, as Fig 5, E12 shows.

Table 11 shows the contrast of prediction regarding COVID and sex interaction. When **COVID-19 periods and sex** are considered together, small differences found for evaluation before COVID-19 disappear during and after the lockdown period. Before COVID-19, the difference between the prediction of being evaluated is significant (-0.029), but after the event, there are no significant differences. In contrast, while during the COVID-19 lockdown period, differences in predictive means of passing and scores between men and women decrease and are nonsignificant, differences for before and after the lockdown period are significant. However, after the lockdown period, they increase in comparison to pre-COVID-19 levels. Both men’s and women’s expected results (passing and scores) worsened, but women’s underperformance increased and again became statistically significant. Their difference in predictive margins was worse at -0.182, while it was only -0.086 before COVID-19. Moreover, the differences in expected scores were the highest of the whole set of variables studied. Women were expected to obtain 0.992 fewer points than men over the postlockdown period, doubling the pre-COVID-19 difference of -0.429 points.

**Table 11:** Contrast of predictions. COVID and sex interactions.

	Evaluated			Passed			Score		
	chi2	df	Contrast	chi2	df	Contrast	F	df	Contrast
Sex over COVID-19									
(Women vs Men) Pre COVID-19	**6.63** **	**1**	**-0.029**	**30.75** ***	**1**	**-0.086**	**26.37** ***	**1**	**-0.429**
			(0.011)			(0.015)			(0.083)
(Women vs Men) Lockdown	0.32	1	0.011	0.04	1	0.005	0.93	1	-0.139
			(0.021)			(0.027)			(0.144)
(Women vs Men) After lockdown	0.51	1	-0.015	**40.33** ***	**1**	**-0.182**	**47.92** ***	**1**	**-0.992**
			(0.022)			(0.028)			(0.143)
Joint	7.67*	3		63.07***	3		21.96***	3	

Notes: Robust standard errors in parentheses;

*** p< 0.01,

** p< 0.05,

* p< 0.1.

Data in bold are significant variables.

## 5. Discussion

From the above results, we observe that women underperform in distance education on a set of applied economics subjects, especially in the central years of life (30–45). Women may be likely to be evaluated similar to men, but their odds of passing and expected scores are lower. To up to 60 years of age, women clearly underperform.

These results support Richardson *et al*.*’s* claim that women’s underperformance happens in their central age period [24] and especially in the first term, when the Christmas holiday occurs before exams. As women care for children more than men, when students are more likely to have small children, gender differences clearly impact academic performance, as is observed in the labour market [36]. Since almost half of the students of both sexes in our sample are between 30 and 45 years of age, this asymmetry can have a major impact on women’s outcomes. Assuming that male UNED students behave similarly to average Spanish men [37, 38], they likely take care of their children less than female UNED students. Accordingly, they have more time in general and especially during school breaks such as the Christmas holiday, which lasts two weeks in Spain. As a result, men can study longer hours and obtain better results, even though both women and men decide to take exams equally. Nonetheless, the Christmas break should have a more positive effect for both sexes in the first term than in the second, as in the latter there is no holiday before exams. In addition, as exams are taken at the end of a term, the accumulated fatigue from caring for children and working and studying could play a role in women’s underperformance. This could explain the significance of the variable term.

Furthermore, the fact that not all women underperform strengthens our conclusions: older students, men and women have the best results, and there is no statistically significant gender gap. Among the causes of better results among older students are deeper learning styles [9] but also fewer conflicts between occupational and family responsibilities and education commitments.

The nationality variable for foreign students does not offer significant results, although its presence in the sample (4% of the observations) is limited. On the other hand, the participation variables confirm its importance in terms of passing a course.

Regarding COVID-19, there is some evidence of better results occurring during the lockdown period, but this is not found in the postlockdown period, in line with the literature assuming that such improvement is due to better time management and involvement in subjects [10] and not due to softer standards due to online exams, as this method of evaluation was maintained in the postlockdown period, when academic results worsen consistently, especially for women.

The greater underperformance of women in the postlockdown period could be attributed to different causes, such as increased domestic and care responsibilities once people can move more freely. The literature shows that women assumed a higher proportion of tasks due to a lack of education services from September 2020 to June 2021 [21], eventually contributing to the increased difference in the postlockdown period. In summary, the situation in the postlockdown period is far from reflecting the pre-COVID-19 period, which explains the worsening learning outcomes for both sexes and the increasing underperformance of women. Men may return sooner, and they are closer to normality, as they do not bear the burden of household and family chores.

One of the main limitations of the paper lies in a lack of control variables, such as being married and the number of children, used as a proxy of care burdens. Such data were not collected either by the university in the administrative enrolment process or by teaching staff. As a result, these variables are missing from our sample. The main finding of the paper (the underperformance of middle-aged women in distance education in economics) is clearly compatible with the argument that women bear the brunt of domestic and care needs and shows a worsening of this situation due to the lockdown period. However, further research based on ad hoc data should be performed to analytically test this hypothesis.

## 6. Conclusions

We found evidence of gender gaps in distance learning in applied economics at UNED. In our sample, women clearly underperform. Age, the field of education, and other academic and structural factors are key variables for understanding where and why men still outperform. This article provides evidence of the existence of gender gaps in higher education on several dimensions.

First, the field of knowledge seems to be relevant to understanding these gaps, as they do not occur homogeneously. Aggregated UNED data show differences between fields of knowledge and degrees, as we have seen in Table 1. Besides, this article finds sound evidence that women underperform in economics, not only in enrolment percentages and the achievement of degrees [45, 46], but also in pass rates and scores.

Second, regarding gender intersectionality, age and term variables clearly affect women’s results, especially when students are in the central years of life (30–45). At this age, students usually have work and family responsibilities; the latter are mainly taken on by women, and could probably result in lower academic performance. In addition, women’s underperformance is considerably worse in the first term. The two-week Christmas holidays are a possible explanation for this fact as children are at home just one month before exams. When the distribution of family and care tasks is not balanced by sex, men have more time to study, while women have less.

Third, the former conclusion has deep implications for higher education distance learning. As the average distance learning student is usually older than his or her in-class counterpart, gender gaps may appear more easily and be more significant. In fact, these gaps may replicate those of the labour market, as one of the most important underlying factors is the same asymmetry in the distribution of household and family chores.

Fourth, the academic consequences of the COVID-19 period have affected students differently by sex, as previous literature has shown. Over the lockdown period, both sexes slightly improved their results. Nevertheless, after this period, women’s results worsened in comparison to those of the pre-COVID-19 period to a greater extent than for men. This could be explained in relation to care and domestic needs as Richardson *et al*. (1999) suggested two decades ago [24]. During the lockdown period, care needs were higher due to the closing of educational centres and the inability to outsource, and more men were present at home, slightly increasing their participation in meeting these needs. While most of the burden fell on women, who were already doing most of the housework before the lockdown [19], sharing some tasks helped reduce the gender gap in learning outcomes. However, once the lockdown passed, men returned to work as in the pre-COVID-19 period and reduced their presence and participation at home, while care and domestic needs increased due to temporal and partial lockdowns, work from home and the maintenance of the previous gendered distribution of care and household chores [21].

Finally, from a policy perspective, some lessons can be learned. First, online exams are not the cause of higher scores, as UNED maintained this type of evaluation in the postlockdown period, but scores worsened. Thus, the use of online exams could be considered academically acceptable. Second, flexibility regarding dates of exams, which were reduced at UNED over the lockdown and postlockdown periods, may impact differentially by sex. As women usually take on more tasks than men, they need more flexibility; as a result, mechanisms to restore or increase flexibility could reduce the gender gap. In addition, flexibility is not only needed on final test dates; more flexibility related to taking CA tests could also result in less gender bias. Third, continuous assessment does not seem to be the obvious means to accommodate both family and school responsibilities: if mandatory tasks increase beyond CA and final exams, the gender gap in nonface-to face higher education could be widened. Finally, given the greater gender differences in academic results over the first term and higher scores for both sexes relative to the second term, degree programs may consider this information to structure content and time allocation to improve students’ results in an ungendered way in the medium and long terms.

Regarding future research, it would be interesting to analyse key factors of the learning-teaching process, as well as sociolabour and family conditions, to explain and contrast the causes of the gender gap found in our students. In addition, analyses could be applied to other faculties with similar gender gaps in global educational outcomes, such as psychology, political science and sociology (see Table 1).

However, if women’s underperformance is due to less participation from men in care and domestic tasks, this trend should be persistent. Thus, further research on the relation between gender gaps at work or in academic results and family tasks is needed to support policy reforms and measures that increase men’s participation in domestic and care needs. The improvement of work-life balance and of men’s involvement at home and with family needs would reduce gender gaps not only at home and in the labour market [21] but also in higher education, especially for middle-aged people.

Work-life balance problems related to a lack of time and double shifts are especially relevant for women in university studies [21]. A lack of time to study at a distance university is also likely a gendered issue related to the sexual division of labour, similar to the different gender gaps found in the labour market. Thus, beyond university changes and improvements, structural policy reforms such as universal early childhood education and long-term care services are needed to reduce gender gaps in higher education when students have labour and family responsibilities [21, 58].

Similarly, gender gaps in economics learning outcomes seem to point to a more structural issue related to gender inequality in education and society and to how gender imbalances are socially perceived and transmitted. On the one hand, inequality in education is focused on where women are less present, such as the underrepresentation of women in STEM or in the labour market, while the underrepresentation of men in some fields, such as education or care and domestic work, is not systematically considered a problem [59]. On the other hand, generalizations erase the nuances and subtleties of gender inequalities, where gender intersectional analysis is key and brings awareness of the origins of gender imbalances. Women do not perform better or worse than men at all stages of the education system or in all branches [60]. Gender inequality persists in many aspects of education [25], and this article highlights this issue; furthermore, gendered student performance could also be seen as an indicator of a deeper inequality social problem.

## Supporting information

S1 Table Evaluated models’ estimation results. (DOCX)

S2 Table Passed models’ estimation results. (DOCX)

S3 Table Score models’ estimation results. (DOCX)

S4 Table Contrast of predictions. (DOCX)

S1 Data (XLS)
