# Supplementary material for: What is behind the gender gap in economics distance education: Age, work-life balance and COVID-19
Source: PLoS One. 2022 Aug 8;17(8):e0272341. doi: 10.1371/journal.pone.0272341 (PMC9359611; doi:10.1371/journal.pone.0272341)
Supplement: S1 Table — (DOCX) [file pone.0272341.s001.docx]

# Supporting information

Table S1. Evaluated models’ estimation results

|  |  | Sociodemographic | | | | |  | Whole-single | | | | |  | Whole-interact | | | | |
| --- | --- | --- | --- | --- | --- | --- | --- | --- | --- | --- | --- | --- | --- | --- | --- | --- | --- | --- |
|  |  | Logit |  | O.R. |  | % |  | Logit |  | O.R. |  | % |  | Logit |  | O.R. |  | % |
|  |  |  |  |  |  |  |  |  |  |  |  |  |  |  |  |  |  |  |
| Women |  | 0.087* |  | 1.091* |  | 9.1 |  | -0.114** |  | 0.893** |  | -10.7 |  | 0.221 |  | 1.248 |  | 24.8 |
|  |  | (0.046) |  | (0.996 - 1.194) |  |  |  | (0.051) |  | (0.807 - 0.987) |  |  |  | (0.208) |  | (0.830 - 1.875) |  |  |
| Age |  | -0.002 |  | 0.998 |  | -0.2 |  | -0.009*** |  | 0.991*** |  | -0.9 |  | **-0.005*** |  | **0.995*** |  | **-0.5** |
|  |  | (0.002) |  | (0.994 - 1.003) |  |  |  | (0.003) |  | (0.986 - 0.996) |  |  |  | (0.003) |  | (0.989 - 1.001) |  |  |
| Women#Age |  |  |  |  |  |  |  |  |  |  |  |  |  | **-0.012**** |  | **0.988**** |  | **-1.2** |
|  |  |  |  |  |  |  |  |  |  |  |  |  |  | (0.005) |  | (0.977 - 0.998) |  |  |
| Foreign |  | -0.036 |  | 0.964 |  | -3.6 |  | 0.061 |  | 1.063 |  | 6.3 |  | **-0.266*** |  | **0.766*** |  | **-23.4** |
|  |  | (0.103) |  | (0.788 - 1.180) |  |  |  | (0.109) |  | (0.858 - 1.317) |  |  |  | (0.154) |  | (0.567 - 1.037) |  |  |
| Women#Foreign |  |  |  |  |  |  |  |  |  |  |  |  |  | **0.521**** |  | **1.684**** |  | **68.4** |
|  |  |  |  |  |  |  |  |  |  |  |  |  |  | (0.213) |  | (1.111 - 2.555) |  |  |
| Second Term |  |  |  |  |  |  |  | -0.561*** |  | 0.571*** |  | -42.9 |  | **-0.589***** |  | **0.555***** |  | **-44.5** |
|  |  |  |  |  |  |  |  | (0.044) |  | (0.523 - 0.623) |  |  |  | (0.052) |  | (0.501 - 0.614) |  |  |
| Women#Second Term |  |  |  |  |  |  |  |  |  |  |  |  |  | 0.098 |  | 1.103 |  | 10.3 |
|  |  |  |  |  |  |  |  |  |  |  |  |  |  | (0.083) |  | (0.936 - 1.299) |  |  |
| Degree_Business Admin. |  |  |  |  |  |  |  | 0.252*** |  | 1.287*** |  | 28.7 |  | **0.245***** |  | **1.278***** |  | **27.8** |
|  |  |  |  |  |  |  |  | (0.055) |  | (1.155 - 1.434) |  |  |  | (0.055) |  | (1.147 - 1.424) |  |  |
| Degree_Tourism |  |  |  |  |  |  |  | 0.808*** |  | 2.243*** |  | 124.3 |  | **0.829***** |  | **2.292***** |  | **129.2** |
|  |  |  |  |  |  |  |  | (0.073) |  | (1.944 - 2.587) |  |  |  | (0.075) |  | (1.980 - 2.653) |  |  |
| Degree_Political Sci. |  |  |  |  |  |  |  | 0.080 |  | 1.084 |  | 8.4 |  | 0.088 |  | 1.092 |  | 9.2 |
|  |  |  |  |  |  |  |  | (0.172) |  | (0.774 - 1.518) |  |  |  | (0.175) |  | (0.776 - 1.539) |  |  |
| CA Test |  |  |  |  |  |  |  | 1.798*** |  | 6.036*** |  | 503.6 |  | **1.796***** |  | **6.028***** |  | **502.8** |
|  |  |  |  |  |  |  |  | (0.051) |  | (5.462 - 6.670) |  |  |  | (0.051) |  | (5.455 - 6.660) |  |  |
| Messages |  |  |  |  |  |  |  | 0.271*** |  | 1.312*** |  | 31.2 |  | **0.272***** |  | **1.313***** |  | **31.3** |
|  |  |  |  |  |  |  |  | (0.035) |  | (1.224 - 1.406) |  |  |  | (0.035) |  | (1.225 - 1.406) |  |  |
| Lockdown |  |  |  |  |  |  |  | 0.981*** |  | 2.668*** |  | 166.8 |  | **0.926***** |  | **2.525***** |  | **152.5** |
|  |  |  |  |  |  |  |  | (0.062) |  | (2.363 - 3.013) |  |  |  | (0.077) |  | (2.170 - 2.937) |  |  |
| After lockdown |  |  |  |  |  |  |  | 0.973*** |  | 2.646*** |  | 164.6 |  | **0.932***** |  | **2.541***** |  | **154.1** |
|  |  |  |  |  |  |  |  | (0.069) |  | (2.309 - 3.031) |  |  |  | (0.087) |  | (2.143 - 3.012) |  |  |
| Women#Lockdown |  |  |  |  |  |  |  |  |  |  |  |  |  | 0.172 |  | 1.187 |  | 18.7 |
|  |  |  |  |  |  |  |  |  |  |  |  |  |  | (0.129) |  | (0.922 - 1.529) |  |  |
| Women#After lockdown |  |  |  |  |  |  |  |  |  |  |  |  |  | 0.115 |  | 1.122 |  | 12.2 |
|  |  |  |  |  |  |  |  |  |  |  |  |  |  | (0.143) |  | (0.848 - 1.485) |  |  |
| Constant |  | -0.049 |  | 0.952 |  |  |  | -0.321*** |  | 0.726*** |  |  |  | **-0.423***** |  | **0.655***** |  |  |
|  |  | (0.090) |  | (0.798 - 1.137) |  |  |  | (0.104) |  | (0.592 - 0.889) |  |  |  | (0.124) |  | (0.514 - 0.836) |  |  |
|  |  |  |  |  |  |  |  |  |  |  |  |  |  |  |  |  |  |  |
| N |  | 16766 |  | 16766 |  |  |  | 16766 |  | 16766 |  |  |  | 16766 |  | 16766 |  |  |
| ll |  | -11606 |  | -11606 |  |  |  | -9686 |  | -9686 |  |  |  | -9673 |  | -9673 |  |  |
| chi2 |  | 4.102 |  | 4.102 |  |  |  | 2142 |  | 2142 |  |  |  | 2176 |  | 2176 |  |  |
| df_model |  | 3 |  | 3 |  |  |  | 11 |  | 11 |  |  |  | 16 |  | 16 |  |  |
| p |  | 0.251 |  | 0.251 |  |  |  | 0.000 |  | 0.000 |  |  |  | 0.000 |  | 0.000 |  |  |
| N_clusters |  | 7449 |  | 7449 |  |  |  | 7449 |  | 7449 |  |  |  | 7449 |  | 7449 |  |  |

Notes: robust standard errors in parentheses; *** p< 0.01, ** p< 0.05, * p< 0.1. Data in bold are significant variables discussed in the text.
