# Supplementary material for: What is behind the gender gap in economics distance education: Age, work-life balance and COVID-19
Source: PLoS One. 2022 Aug 8;17(8):e0272341. doi: 10.1371/journal.pone.0272341 (PMC9359611; doi:10.1371/journal.pone.0272341)
Supplement: S2 Table — (DOCX) [file pone.0272341.s002.docx]

# Supporting information

Table S2. Passed models’ estimation results

|  |  | Sociodemographic | | | | |  | Whole-single | | | | |  | Whole-interact | | | | |
| --- | --- | --- | --- | --- | --- | --- | --- | --- | --- | --- | --- | --- | --- | --- | --- | --- | --- | --- |
|  |  | Logit |  | O.R. |  | % |  | Logit |  | O.R. |  | **%** |  | Logit |  | O.R. |  | % |
|  |  |  |  |  |  |  |  |  |  |  |  |  |  |  |  |  |  |  |
| Women |  | -0.333*** |  | 0.717*** |  | -28.3 |  | -0.401*** |  | 0.670*** |  | -33 |  | **-0.542**** |  | **0.582**** |  | **-41.8** |
|  |  | (0.057) |  | (0.641 - 0.802) |  |  |  | (0.061) |  | (0.594 - 0.755) |  |  |  | (0.243) |  | (0.361 - 0.937) |  |  |
| Age |  | 0.023*** |  | 1.024*** |  | 2.4 |  | 0.019*** |  | 1.019*** |  | 1.9 |  | **0.019***** |  | **1.019***** |  | **1.9** |
|  |  | (0.003) |  | (1.018 - 1.030) |  |  |  | (0.003) |  | (1.013 - 1.025) |  |  |  | (0.004) |  | (1.011 - 1.026) |  |  |
| Women#Age |  |  |  |  |  |  |  |  |  |  |  |  |  | 0.000 |  | 1.000 |  | 0 |
|  |  |  |  |  |  |  |  |  |  |  |  |  |  | (0.006) |  | (0.988 - 1.013) |  |  |
| Foreign |  | -0.084 |  | 0.919 |  | -8.1 |  | -0.046 |  | 0.955 |  | -4.5 |  | -0.081 |  | 0.922 |  | -7.8 |
|  |  | (0.132) |  | (0.710 - 1.190) |  |  |  | (0.138) |  | (0.729 - 1.252) |  |  |  | (0.246) |  | (0.570 - 1.493) |  |  |
| Women#Foreign |  |  |  |  |  |  |  |  |  |  |  |  |  | 0.024 |  | 1.024 |  | 2.4 |
|  |  |  |  |  |  |  |  |  |  |  |  |  |  | (0.297) |  | (0.572 - 1.835) |  |  |
| Second Term |  |  |  |  |  |  |  | -0.174** |  | 0.840** |  | -16 |  | -0.265*** |  | **0.767***** |  | **-23.3** |
|  |  |  |  |  |  |  |  | (0.069) |  | (0.734 - 0.962) |  |  |  | (0.081) |  | (0.655 - 0.899) |  |  |
| Women#Second Term |  |  |  |  |  |  |  |  |  |  |  |  |  | **0.307**** |  | **1.359**** |  | **35.9** |
|  |  |  |  |  |  |  |  |  |  |  |  |  |  | (0.120) |  | (1.075 - 1.718) |  |  |
| Degree_Business Admin. |  |  |  |  |  |  |  | -0.430*** |  | 0.650*** |  | -35 |  | **-0.459***** |  | **0.632***** |  | **-36.8** |
|  |  |  |  |  |  |  |  | (0.076) |  | (0.561 - 0.754) |  |  |  | (0.075) |  | (0.545 - 0.732) |  |  |
| Degree_Tourism |  |  |  |  |  |  |  | 0.175** |  | 1.192** |  | 19.2 |  | 0.249*** |  | **1.283***** |  | **28.3** |
|  |  |  |  |  |  |  |  | (0.083) |  | (1.013 - 1.402) |  |  |  | (0.086) |  | (1.083 - 1.519) |  |  |
| Degree_Political Sci. |  |  |  |  |  |  |  | 0.511** |  | 1.667** |  | 66.7 |  | **0.513**** |  | **1.670**** |  | **67** |
|  |  |  |  |  |  |  |  | (0.258) |  | (1.006 - 2.762) |  |  |  | (0.259) |  | (1.006 - 2.775) |  |  |
| CA Test |  |  |  |  |  |  |  | 0.889*** |  | 2.433*** |  | 143.3 |  | **0.886***** |  | **2.426***** |  | **142.6** |
|  |  |  |  |  |  |  |  | (0.053) |  | (2.192 - 2.700) |  |  |  | (0.053) |  | (2.185 - 2.693) |  |  |
| Messages |  |  |  |  |  |  |  | 0.094*** |  | 1.099*** |  | 9.9 |  | **0.095***** |  | **1.100***** |  | **10** |
|  |  |  |  |  |  |  |  | (0.022) |  | (1.053 - 1.146) |  |  |  | (0.022) |  | (1.054 - 1.147) |  |  |
| Lockdown |  |  |  |  |  |  |  | 0.337*** |  | 1.401*** |  | 40.1 |  | **0.237**** |  | **1.267**** |  | **26.7** |
|  |  |  |  |  |  |  |  | (0.082) |  | (1.193 - 1.644) |  |  |  | (0.102) |  | (1.038 - 1.547) |  |  |
| After lockdown |  |  |  |  |  |  |  | -0.600*** |  | 0.549*** |  | -45.1 |  | **-0.488***** |  | **0.614***** |  | **-38.6** |
|  |  |  |  |  |  |  |  | (0.072) |  | (0.476 - 0.632) |  |  |  | (0.095) |  | (0.509 - 0.740) |  |  |
| Women#Lockdown |  |  |  |  |  |  |  |  |  |  |  |  |  | 0.250 |  | 1.285 |  | 28.5 |
|  |  |  |  |  |  |  |  |  |  |  |  |  |  | (0.166) |  | (0.928 - 1.778) |  |  |
| Women#After lockdown |  |  |  |  |  |  |  |  |  |  |  |  |  | **-0.272*** |  | **0.762*** |  | **-23.8** |
|  |  |  |  |  |  |  |  |  |  |  |  |  |  | (0.146) |  | (0.572 - 1.015) |  |  |
| Constant |  | -0.148 |  | 0.863 |  |  |  | -0.219* |  | 0.804* |  |  |  | -0.170 |  | 0.844 |  |  |
|  |  | (0.115) |  | (0.689 - 1.081) |  |  |  | (0.124) |  | (0.631 - 1.024) |  |  |  | (0.154) |  | (0.623 - 1.142) |  |  |
|  |  |  |  |  |  |  |  |  |  |  |  |  |  |  |  |  |  |  |
| N |  | 8082 |  | 8082 |  |  |  | 8082 |  | 8082 |  |  |  | 8082 |  | 8082 |  |  |
| ll |  | -5228 |  | -5228 |  |  |  | -4961 |  | -4961 |  |  |  | -4948 |  | -4948 |  |  |
| chi2 |  | 101.3 |  | 101.3 |  |  |  | 564.1 |  | 564.1 |  |  |  | 595.1 |  | 595.1 |  |  |
| df_model |  | 3 |  | 3 |  |  |  | 11 |  | 11 |  |  |  | 16 |  | 16 |  |  |
| p |  | 0.000 |  | 0.000 |  |  |  | 0.000 |  | 0.000 |  |  |  | 0.000 |  | 0.000 |  |  |
| N_clusters |  | 4544 |  | 4544 |  |  |  | 4544 |  | 4544 |  |  |  | 4544 |  | 4544 |  |  |

Notes: robust standard errors in parentheses; *** p< 0.01, ** p< 0.05, * p< 0.1. Data in bold are significant variables discussed in the text.
