# Supplementary material for: What is behind the gender gap in economics distance education: Age, work-life balance and COVID-19
Source: PLoS One. 2022 Aug 8;17(8):e0272341. doi: 10.1371/journal.pone.0272341 (PMC9359611; doi:10.1371/journal.pone.0272341)
Supplement: S3 Table — (DOCX) [file pone.0272341.s003.docx]

# Supporting information

Table S3. Score models’ estimation results

|  |  | Sociodemographic |  | Whole-single |  | Whole-interact |
| --- | --- | --- | --- | --- | --- | --- |
|  |  | Coef. |  | Coef. |  | Coef. |
|  |  |  |  |  |  |  |
| Women |  | -0.423*** |  | -0.470*** |  | **-0.552**** |
|  |  | (0.072) |  | (0.071) |  | (0.275) |
| Age |  | 0.029*** |  | 0.022*** |  | **0.021***** |
|  |  | (0.004) |  | (0.003) |  | (0.004) |
| Women#Age |  |  |  |  |  | 0.000 |
|  |  |  |  |  |  | (0.007) |
| Foreign |  | -0.242 |  | -0.178 |  | -0.279 |
|  |  | (0.156) |  | (0.152) |  | (0.267) |
| Women#Foreign |  |  |  |  |  | 0.120 |
|  |  |  |  |  |  | (0.325) |
| Second Term |  |  |  | -0.417*** |  | **-0.481***** |
|  |  |  |  | (0.071) |  | (0.084) |
| Women#Second Term |  |  |  |  |  | **0.239*** |
|  |  |  |  |  |  | (0.132) |
| Degree_Business Admin. |  |  |  | -0.532*** |  | **-0.559***** |
|  |  |  |  | (0.088) |  | (0.088) |
| Degree_Tourism |  |  |  | 0.180** |  | **0.243***** |
|  |  |  |  | (0.088) |  | (0.090) |
| Degree_Political Sci. |  |  |  | 0.368 |  | 0.358 |
|  |  |  |  | (0.274) |  | (0.270) |
| CA Test |  |  |  | 1.132*** |  | **1.128***** |
|  |  |  |  | (0.060) |  | (0.060) |
| Messages |  |  |  | 0.081*** |  | **0.082***** |
|  |  |  |  | (0.011) |  | (0.011) |
| Lockdown |  |  |  | 0.529*** |  | **0.474***** |
|  |  |  |  | (0.089) |  | (0.115) |
| After lockdown |  |  |  | -0.661*** |  | **-0.476***** |
|  |  |  |  | (0.079) |  | (0.101) |
| Women#Lockdown |  |  |  |  |  | 0.150 |
|  |  |  |  |  |  | (0.179) |
| Women#After lockdown |  |  |  |  |  | **-0.461***** |
|  |  |  |  |  |  | (0.162) |
| Constant |  | 4.265*** |  | 4.219*** |  | **4.247***** |
|  |  | (0.142) |  | (0.141) |  | (0.171) |
|  |  |  |  |  |  |  |
| N |  | 8082 |  | 8082 |  | 8082 |
| R-squared |  | 0.023 |  | 0.106 |  | 0.109 |
| r2_a |  | 0.0231 |  | 0.105 |  | 0.107 |
| F |  | 38.69 |  | 75.35 |  | 54.07 |
| df_model |  | 3 |  | 11 |  | 16 |
| p |  | 0.000 |  | 0.000 |  | 0.000 |
| N_clusters |  | 4544 |  | 4544 |  | 4544 |

Notes: robust standard errors in parentheses; *** p< 0.01, ** p< 0.05, * p< 0.1. Data in bold are significant variables discussed in the text.
