# Supplementary material for: What is behind the gender gap in economics distance education: Age, work-life balance and COVID-19
Source: PLoS One. 2022 Aug 8;17(8):e0272341. doi: 10.1371/journal.pone.0272341 (PMC9359611; doi:10.1371/journal.pone.0272341)
Supplement: S4 Table — (DOCX) [file pone.0272341.s004.docx]

# Supporting information

**Table S4. Contrast of predictions**.

|  |  |  | Evaluated | | | | | | |  |  | Passed | | | | | | |  |  | Score | | | | | | |
| --- | --- | --- | --- | --- | --- | --- | --- | --- | --- | --- | --- | --- | --- | --- | --- | --- | --- | --- | --- | --- | --- | --- | --- | --- | --- | --- | --- |
|  |  | chi2 | df | P>chi2 | Contrast | Std. Err. | C.I. (95%) | | |  | chi2 | df | P>chi2 | Contrast | Std. Err. | C.I. (95%) | | |  | F | df | P>F | Contrast | Std. Err. | C.I. (95%) | | |
| Sex over nationality |  |  |  |  |  |  |  |  |  |  |  |  |  |  |  |  |  |  |  |  |  |  |  |  |  |  |  |
| (Women vs Men) Spanish |  | **7.53***** | 1 | 0.006 | **-.028** | .010 | (-.048 | ; | -.008) |  | **43.13***** | 1 | 0.000 | **-.088** | .013 | (-.114 | ; | -.061) |  | **44.42***** | 1 | 0.000 | **-.481** | .072 | (-.622 | ; | -.339) |
| (Women vs Men) Foreign |  | 3.83* | 1 | 0.050 | **.080** | .041 | (-.000 | ; | .161) |  | 1.72 | 1 | 0.189 | -.085 | .064 | (-.212 | ; | .042) |  | 1.17 | 1 | 0.280 | -.342 | .316 | (-.963 | ; | .279) |
| Joint |  | **11.56***** | 2 | 0.003 |  |  |  |  |  |  | **44.51***** | 2 | 0.000 |  |  |  |  |  |  | **22.67***** | 2 | 0.000 |  |  |  |  |  |
|  |  |  |  |  |  |  |  |  |  |  |  |  |  |  |  |  |  |  |  |  |  |  |  |  |  |  |  |
| Sex over term |  |  |  |  |  |  |  |  |  |  |  |  |  |  |  |  |  |  |  |  |  |  |  |  |  |  |  |
| (Women vs Men) First term |  | **6.87***** | 1 | 0.008 | **-.035** | .013 | (-.061 | ; | -.008) |  | **57.35***** | 1 | 0.000 | **-.131** | .017 | (-.165 | ; | -.097) |  | **54.77***** | 1 | 0.000 | **-.655** | .088 | (-.829 | ; | -.482) |
| (Women vs Men) Second term |  | 1.09 | 1 | 0.295 | -.011 | .011 | (-.034 | ; | .010) |  | 3.51 | 1 | 0.061 | -.0316 | .016 | (-.064 | ; | .001) |  | **7.02***** | 1 | 0.008 | **-.241** | .091 | (-.419 | ; | -.062) |
| Joint |  | **6.94**** | 2 | 0.031 |  |  |  |  |  |  | **57.72***** | 2 | 0.000 |  |  |  |  |  |  | **27.85***** | 2 | 0.000 |  |  |  |  |  |
|  |  |  |  |  |  |  |  |  |  |  |  |  |  |  |  |  |  |  |  |  |  |  |  |  |  |  |  |
| Sex over COVID-19 |  |  |  |  |  |  |  |  |  |  |  |  |  |  |  |  |  |  |  |  |  |  |  |  |  |  |  |
| (Women vs Men) Pre COVID-19 |  | **6.63**** | 1 | 0.010 | **-.029** | .011 | (-.051 | ; | -.006) |  | **30.75***** | 1 | 0.000 | **-.086** | .015 | (-.116 | ; | -.055) |  | **26.37***** | 1 | 0.000 | **-.429** | .083 | (-.593 | ; | -.265) |
| (Women vs Men) Lockdown |  | 0.32 | 1 | 0.572 | .011 | .021 | (-.029 | ; | .053) |  | 0.04 | 1 | 0.846 | .005 | .027 | (-.048 | ; | .058) |  | 0.93 | 1 | 0.333 | -.139 | .144 | (-.424 | ; | .144) |
| (Women vs Men) After lockdown |  | 0.51 | 1 | 0.477 | -.015 | .022 | (-.059 | ; | .027) |  | **40.33***** | 1 | 0.000 | **-.182** | .028 | (-.239 | ; | -.126) |  | **47.92***** | 1 | 0.000 | **-.992** | .143 | (-1.273 | ; | -.711) |
| Joint |  | **7.67*** | 3 | 0.053 |  |  |  |  |  |  | **63.07***** | 3 | 0.000 |  |  |  |  |  |  | **21.96***** | 3 | 0.000 |  |  |  |  |  |
|  |  |  |  |  |  |  |  |  |  |  |  |  |  |  |  |  |  |  |  |  |  |  |  |  |  |  |  |

Note: Contrast of predictive margins for evaluated and passed models and linear prediction for score models. Data in bold are significant variables discussed in the text
